# Coptisine Inhibits Influenza Virus Replication by Upregulating p21

**DOI:** 10.3390/molecules28145398

**Published:** 2023-07-14

**Authors:** Ming-Feng He, Jian-Hui Liang, Yan-Ni Shen, Chao-Wei Zhang, Kuang-Yang Yang, Li-Chu Liu, Qian Xie, Chun Hu, Xun Song, Yan Wang

**Affiliations:** 1 Foshan Hospital of Traditional Chinese Medicine, Foshan 528000, China; hemf@fshtcm.com.cn (M.-F.H.); yangky@fshtcm.com.cn (K.-Y.Y.); liulc@fshtcm.com.cn (L.-C.L.); 2 Center for Translation Medicine Research and Development, Shenzhen Institutes of Advanced Technology, Chinese Academy of Sciences, Shenzhen 518055, China; jh.liang@siat.ac.cn (J.-H.L.); yn.shen@siat.ac.cn (Y.-N.S.); qian.xie@szu.edu.cn (Q.X.); 3 Key Laboratory of Structure-Based Drug Design & Discovery, Ministry of Education, School of Pharmaceutical Engineering, Shenyang Pharmaceutical University, Shenyang 110016, China; chunhu@syphu.edu.cn; 4 School of Pharmaceutical Science, Shenzhen University, Shenzhen 518000, China; 2110245018@email.szu.edu.cn; 5 College of Pharmacy, Shenzhen Technology University, Shenzhen 518118, China

**Keywords:** coptisine, influenza virus, antiviral, p21

## Abstract

The activation of innate antiviral immunity is a promising approach for combatting viral infections. In this study, we screened Chinese herbs that activated human immunity and identified coptisine as a potent inhibitor of the influenza virus with an EC_50_ of 10.7 μM in MDCK cells. The time of an addition assay revealed that pre-treatment with coptisine was more effective at reducing viral replication than co-treatment or post-treatment. Our bulk RNA-sequencing data showed that coptisine upregulated the p21 signaling pathway in MDCK cells, which was responsible for its antiviral effects. Specifically, coptisine increased the expression of p21 and FOXO1 in a dose-dependent manner while leaving the MELK expression unchanged. Docking analysis revealed that coptisine likely inhibited MELK activity directly by forming hydrogen bonds with ASP-150 and GLU-87 in the catalytic pocket. These findings suggest that coptisine may be a promising antiviral agent that regulates the p21 signaling pathway to inhibit viral replication.

## 1. Introduction

The influenza virus, as a member of the Orthomyxoviridae family [1], cause acute respiratory infections in humans and are responsible for 300,000–500,000 deaths each year worldwide, with a mortality rate of approximately 0.3–0.5% [2]. These viruses can be divided into four types (A, B, C, and D) based on their core proteins [3,4]. Seasonal influenza viruses that circulate in the population are typically Type A (H1N1 and H3N2 subtypes) and Type B (Yamagata and Victoria strains). Influenza A virus (IAV) has a large number of hosts in nature and is more prone to mutations or reassortments, resulting in a rapid spread in the population [5]. While vaccination and antiviral drugs are the primary treatments for influenza, the emergence of viral resistance [6,7] and adverse side effects [8,9] have made the discovery of new antiviral drugs a pressing concern.

The principle of action for synthetic antiviral drugs is mainly to target enzymes or proteins in relation to replication and release in the virus [10,11,12], thereby preventing the virus from continuing to infect other normal cells. Unlike synthetic drugs, traditional Chinese medicine often indirectly inhibits viruses by activating innate antiviral immunity in the host cells [13,14,15]. Therefore, we screened the effective ingredients of some Chinese herbs that could activate human immunity [16,17], hoping to obtain some phytochemicals with anti-influenza virus activity from them.

**Figure 1:**
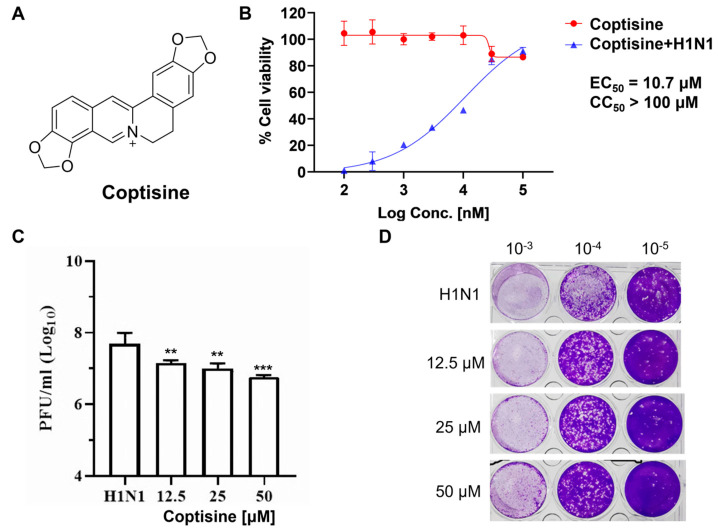
In vitro inhibitory activity of coptisine against H1N1 influenza virus infection. (**A**) Chemical structure of coptisine. (**B**) For the cytopathic effect assay, the H1N1 virus was incubated with coptisine at 4 °C for 30 min before infecting MDCK cells at 37 °C for 2 h. Subsequently, the viral inoculum was removed and replaced by adding coptisine. CPE was observed at 24 h post−incubation by microscopy. For the cytotoxicity test, coptisine was applied to MDCK cells and incubated for 24 h at the indicated concentrations. Then, cell viability was measured by the CCK−8 reagent. The EC_50_ and CC_50_ values were calculated using the inhibitor dose−response function in Prism 5. (**C**) After treating the cells as described in B, the viral titer was detected by a plaque assay and shown as a log value. Bars represent the mean and SD of three biological replicates, with dots representing the values of the replicates. Statistical significance was analyzed with Dunnett’s test (** *p* < 0.01; *** *p* < 0.005, compared with H1N1−treated) (**D**) Representative images of viral plaques in each histogram are shown in (**C**).

In this study, we first identified the anti-IAV activity of coptisine in vitro and elucidated its potential mechanism of action for inhibiting IAV infection. Coptisine (Figure 1A) is a bioactive isoquinoline alkaloid derived from *Coptis chinensis Franch*. The antiviral properties of *Coptis chinensis* have been reported [18], but the bioactive components and pharmacological mechanism need further exploration. We first confirmed the anti-influenza virus activity of coptisine in vitro through a CPE reduction experiment and plaque experiment. Next, we speculated that coptisine could resist influenza infection by activating host immunity and then designed a time addition experiment to confirm our hypothesis. Finally, we elucidated the signaling pathways activated by coptisine at the molecular level through RNA sequencing and a series of biochemical experiments. Our results indicate that coptisine could be used as a MELK inhibitor to manage influenza virus infection.

## 2. Results

### 2.1. Antiviral Activity of Coptisine against H1N1 In Vitro

The influenza virus infection could cause typical cell cytopathic effects (CPE) [1]. To determine the antiviral activity of Coptisine, a combination of Coptisine and the H1N1 virus was inoculated into MDCK cells. After the virus was removed, Coptisine was supplemented for 24 h. The results show that Coptisine significantly reduced CPE formation on MDCK cells induced by an H1N1 virus in a dose-dependent manner, with an EC_50_ of 12.04 μM (Figure 1B).

To further evaluate the anti-influenza virus efficacy of Coptisine, we assessed the effect of Coptisine on the virus titer in the cell supernatant using a plaque reduction assay 48 h after administration. The results showed that the plaque of each group treated with different concentrations of Coptisine was significantly reduced compared to the control group, indicating that Coptisine could reduce H1N1 replication in the MDCK cell (Figure 1C,D). These in vitro experiments demonstrate that Coptisine has significant antiviral efficacy against the H1N1 influenza virus.

### 2.2. Administering Coptisine before Infection Is More Effective in Exerting Its Antiviral Effects

**Figure 2:**
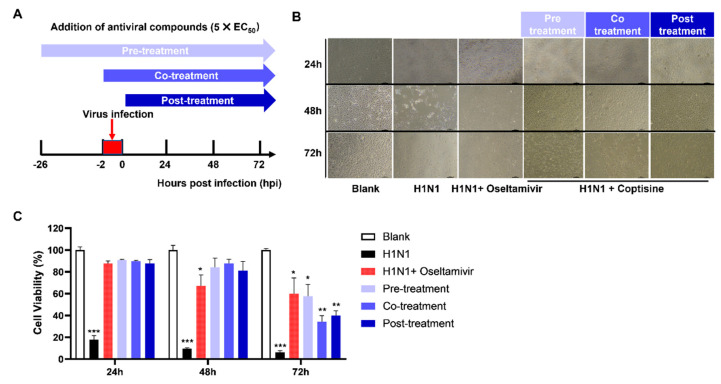
Time−of−addition assay used to identify the steps of the H1N1 virus life cycle, which could be inhibited by coptisine. (**A**) Schematic outline of the time−of−addition assay. MDCK cells were inoculated with H1N1 and treated with ~10 times the EC_50_ of coptisine or oseltamivir. Cell viability was measured at 24, 48, and 72 hpi. (**B**) MDCK cells were infected with H1N1, and oseltamivir or coptisine was added at different time points, as shown in (**A**). Microscopic images of cells were observed at 24, 48, and 72 hpi. (**C**) Measuring cell viability at different time points after treating MDCK cells with different protocols, and the results are presented as histograms. Each bar represents the mean ± SD of three independent experiments. Statistical significance was analyzed with Dunnett’s test (* *p* < 0.05; ** *p* < 0.01; *** *p* < 0.005, compared with blank).

Drawing on the traditional Chinese medical science theory and previous research on TCM’s antiviral mechanisms [19], we postulated that coptisine’s antiviral action involved directly activating the innate antiviral immunity of host cells. We conducted a time-of-addition assay (Figure 2A) using a concentration of 5 × EC_50_ coptisine and designed various medication modes, including pre-treatment, co-treatment, and post-treatment. Our findings demonstrate that coptisine treatment improved the cell viability reduction caused by viral infections at 24-, 48-, and 72 h post-infection (hpi). A microscopic observation (Figure 2B) reveals that the morphology and cell density of coptisine-treated cells were superior to those of the H1N1 infection group at different time points post-infection. The histogram of cell viability (Figure 2C) indicated no significant differences among the three groups at 24 and 48 hpi. However, at 72 h post-infection, the pre-treatment group’s cell viability was significantly higher than the other groups, suggesting that administering coptisine before infection was more effective when exerting its antiviral effects. Taken together, our results suggest that coptisine, when added 24 h before infection, could alleviate the influenza virus infection’s damage and suppress IAV infection, indicating that coptisine might activate the innate antiviral immunity of host cells.

### 2.3. Coptisine Upregulates the Expression of CDKNI1A and FOXO1

**Figure 3:**
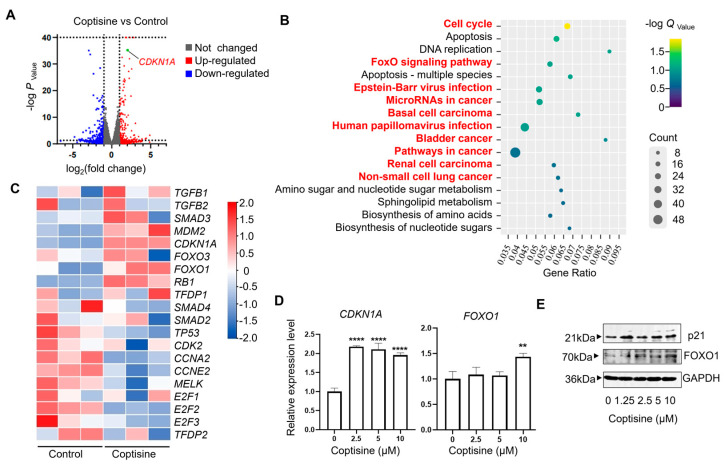
The key genes and signaling pathways modulated by coptisine were explored by bulk RNA sequencing. (**A**) Volcano plot displaying differentially expressed genes between coptisine and control groups (*n* = 3 biological replicates). Significant genes were called via Cuffdiff. The red dots represent the up−regulated expressed transcripts between coptisine and control; the blue dots represent the transcripts whose expression was down−regulated. (**B**) Significantly enriched modulated KEGG pathways. The depth of the color represents the adjusted *p*-value, while the area of the circle represents gene counts. The pathway marked in red font is related to *CDKN1A*. (**C**) Heat map of differential expression of genes associated with *CDKN1A* between the control and coptisine groups (*n* = 3 biologically independent cell samples; FDR < 0.001). Statistical tests were embedded in Cuffdiff. Each row represents a gene; red means an increased gene expression, blue means decreased gene expression, and the darker the color, the more obvious the trend is. Heatmap analysis shows that coptisine could significantly upregulate or downregulate the expression of certain genes compared to the controls. The RNA-Seq data were deposited in GEO (GSE232189) (**D**,**E**) MDCK was cultured for 24 h in the presence of different concentrations of coptisine. The total RNA was isolated to detect the expression level of *CDKN1A* and *FOXO1* by quantitative PCR (**D**). The total protein was collected for Western blot to quantify p21 and FOXO1 (**E**). Each error bar represents the mean ± SD of triplicate wells assayed for each sample. Statistical significance was analyzed with Dunnett’s test (** *p* < 0.01; **** *p* < 0.001, compared with 0 μM).

After confirming that coptisine could resist influenza viruses by activating the host’s innate immune response, we aimed to further explore the relevant genes and signaling pathways involved in the innate immune response against viruses activated by coptisine. Therefore, we performed bulk RNA sequencing to identify the critical genes regulated by coptisine. The volcano plot in Figure 3A displays the distribution of differential gene expressions between the two samples. Among the genes significantly upregulated by coptisine, CDKN1A could be associated with host immunity. The protein p21 encoded by CDKN1A plays a crucial role in maintaining the balance of innate immune activity [20,21]. The study of Ma C et al. also showed that p21 is influenza limiting factor [22]. Figure 3B summarizes the enriched pathways, highlighting pathways related to CDKN1A in red, including the cell cycle, FoxO signaling pathway, Epstein–Barr virus infection, and some cancers. It is worth noting that the FoxO signaling pathway could regulate cellular antiviral response [23]. The heatmap displays that the expression levels of upstream and downstream genes of CDKN1A could be upregulated by coptisine in MDCK cells (Figure 3C). The RNA-Seq data were deposited in GEO (GSE232189). To further demonstrate the above conclusions, we validated the expression of CDKN1A and its upstream regulator, FOXO1, at the mRNA and protein levels. The RT-qPCR results (Figure 3D) show that coptisine significantly upregulated the mRNA expression of CDKN1A at lower concentrations (2.5 μM), but only when the concentration reached 10 μM increased the expression of FOXO1 significantly. The following Western blot (Figure 3E) showed consistent results with RT-qPCR.

### 2.4. Coptisine Might Target against MELK to Inhibit Influenza Virus

MELK (maternal embryonic leucine zipper kinase), a cell cycle-dependent protein kinase belonging to the KIN1/PAR-1/MARK family, is a crucial target of coptisine to inhibit the influenza virus [24]. Previous research has shown that MELK could induce p21 protein expression through the novel substrate FOXO1 after its inhibition [25]. We conducted experiments to determine whether coptisine inhibited MELK expression and found that it did not decrease MELK at the gene and protein levels in MDCK cells treated with coptisine (50 μM) for 24 h (Supplementary Figure S1A,B). However, molecular docking revealed that coptisine could form hydrogen bonds with specific amino acid residues, including ASP-150 and GLU-87, in the catalytic pocket of MELK, indicating coptisine’s interaction with MELK (Supplementary Figure S1C). Therefore, we proposed that coptisine potently inhibited MELK activity, inducing FOXO1 and its downstream p21 expression, which could regulate the host’s immune system to resist the influenza virus infection (Supplementary Figure S1D).

## 3. Discussion

This study aimed to identify active compounds from traditional Chinese medicines that could activate the autoimmune system for antiviral drug development. Our results showed that coptisine exhibited strong anti-influenza virus (H1N1) activities in vitro, as confirmed by CPE reduction and plaque assays. The time of the addition assay revealed that coptisine exerted antiviral activity by activating the host’s innate antiviral immunity. RNA sequencing analysis indicated that coptisine upregulated the expression of *CDKN1A*: a gene related to the host’s immune system. RT-qPCR and Western blot assays further confirmed that coptisine upregulated the expression of CDKN1A and its upstream regulator, FOXO1. Molecular docking results showed that coptisine inhibited MELK activity, an upstream regulator of FOXO1, leading to the upregulation of FOXO1 and p21, and thereby, exerting antiviral activity. In conclusion, our study demonstrated that coptisine, an isoquinoline alkaloid derived from the traditional Chinese herbal medicine *Coptis chinensis Franch*, could effectively improve the host’s antiviral ability by regulating the p21 signaling pathway. According to The Plant List (http://www.theplantlist.org/, accessed on 26 June 2023), there are a total of 13 species in the genus *Coptis*, including *Coptis asplenifolia* Salisb., *Coptis chinensis* Franch., *Coptis deltoidea* C.Y.Cheng and P.K.Hsiao, *Coptis japonica* (Thunb.) Makino, *Coptis laciniata* A.Gray, *Coptis minamitaniana* Kadota, *Coptis occidentalis* (Nutt.) Torr. and A.Gray, *Coptis omeiensis* (C.Chen) C.Y.Cheng, *Coptis quinquefolia* Miq., *Coptis quinquesecta* W.T.Wang, *Coptis teeta* Wall., *Coptis trifolia* (L.) Salisb., and *Coptis trifoliolata* (Makino) Makino. If coptisine can be found in these species, these plants have a good chance of exhibiting antiviral activity through the upregulation of p21 in the host cell.

## 4. Materials and Methods

### 4.1. Cells and Viruses

Madin-Darby canine kidney (MDCK) cells obtained from ATCC were maintained in Dulbecco’s Modified Eagle Medium (DMEM) (Gibco: C11995500BT) and supplemented with 10% inactivated fetal bovine serum (FBS) (Gibco: 10270106) and 1% penicillin/streptomycin (Gibco: 15140-122) at 37 °C and 5% CO_2_. Coptisine was obtained from Alfa Biotechnology Co., Ltd. (Chengdu, China; Lot. AF21091551, purity, >99.5%). The influenza A virus strain A/PR/8/34 was propagated in MDCK cells, and its titer was determined as previously described [16]. The viruses were stored at −80 °C until further use.

### 4.2. Cytotoxicity Assay

MDCK cells were seeded in 96-well culture plates at a density of 1.0 × 10^4^ cells/well and were cultured at 37 °C with 5% CO_2_ overnight. The cells were then treated with different concentrations of coptisine obtained from the National Institutes for Food and Drug Control (Beijing, China) for 24 h. After that, 10 μL of the CCK-8 reagent (Dojindo, Rockville, MD, USA, CK04) was added to each well, followed by 2 h of incubation. The absorbance was measured at 450 nm using a microplate reader (Rayto, Shenzhen, China).

### 4.3. Cytopathic Effect Assay

MDCK cells were seeded in 12-well plates at a density of 2.0 × 10^5^ cells/well and incubated at 37 °C with 5% CO_2_ overnight. The next day, different concentrations of coptisine and A/PR/8/34 (H1N1) (MOI = 0.5) were mixed at 4 °C for 30 min before being added to the MDCK cells at 37 °C for 2 h. After that, the supernatant was removed, and the cells were washed twice with PBS (Solarbio, P1010, Beijing, China). Different concentrations of chrysin were added to the cells, which were then cultured for 24 h at 37 °C. The cytopathic effect was observed under a microscope.

### 4.4. Plaque Reduction Assay

Confluent MDCK cells in 12-well plates were washed with PBS and inoculated with a gradient dilution of the virus solution (MOI = 0.5) [Opti-MEM (Thermo Fisher Scientific, Catalog number: 31985070, Waltham, MA, USA) with 0.3% BSA and 2.5 μg/mL TPCK-treated trypsin (Sigma–Aldrich, Catalog number: T1426, St. Louis, MO, USA)] for 2 h. After removing the virus inoculum, cell monolayers were overlaid with 1.5 mL of the semisolid medium (1.5% CMCNa:DMEM = 1:1, with 0.3% BSA and 2.5 μg/mL TPCK-treated trypsin) and incubated at 37 °C for 30 h. The cell monolayers were then fixed with 4% paraformaldehyde and stained with 0.2% (*w*/*v*) crystal violet (Sigma, C0775). The size of the plaques was counted and measured using Image J software 1.50 version.

### 4.5. RNA Sequencing

To perform RNA sequencing, MDCK cells were initially seeded in a 6-well plate and allowed to incubate for 24 h prior to drug treatment. Subsequently, the cells were collected and sent to the BGI Genomics institution for RNA extraction and bulk mRNA sequencing. Libraries were constructed according to the manufacturer’s instructions (Illumina, San Diego, CA, USA), and paired-end sequencing was conducted using the Illumina Hiseq2000 sequencer (Illumina, USA). Both library construction and RNA-seq were conducted at BGI. The raw sequencing reads (fastq) quality was checked using SOAPnuke (v1.5.2), and alignment was performed by Bowtie2 (v2.2.5). The expression level of genes was calculated using RSEM (v1.2.12), while DESeq2 (v1.4.5) was used for differential expression analysis with a *p*-value < 0.05.

### 4.6. Western Blot Analysis

MDCK cells were treated with drugs according to the protocol and then washed with ice-cold PBS before being lysed with a RIPA buffer (Thermo Scientific, Catalog number: 89900, Waltham, MA, USA) for protein extraction. The extracted protein was quantified using the BCA assay kit (Beyotime, Catalog number: P0012, Shanghai, China) and then mixed with a 5 × protein loading buffer (Meilunbio, Catalog number: MA0003-D, Dalian, China). The samples were boiled at 95 °C for 5 min, and an equivalent quantity of protein samples was loaded onto a 10% sodium dodecyl sulfate-polyacrylamide gel electrophoresis gel (Beyotime, Catalog number: P0012A, Shanghai, China). The protein was subsequently transferred to a polyvinylidene fluoride (PVDF) membrane (Merck Millipore, Catalog number: IPVH15150, Billerica, MA, USA). This nonspecific binding was blocked with a 3% BSA solution, and the membrane was incubated with a primary antibody overnight at 4 °C. The primary antibodies are listed here: Rabbit Anti-MELK antibody (Bioss, Catalog number: bs-12201R, Woburn, MA, USA), Rabbit Anti-p21 antibody (Bioss, Catalog number: bsm-60698R, Woburn, MA, USA), Rabbit Anti-FOXO1 antibody (Bioss, Catalog number: bs-23175R, Woburn, MA, USA), Anti-GAPDH Rabbit polyclonal antibody(Sangon Biotech, Catalog number: D110016, Shanghai, China), and the secondary antibody was the Goat Anti-Rabbit IgG antibody (HRP) (GeneTex, Catalog number: GTX213110, Irvine, CA, USA). After washing with a Tris-buffered solution containing 0.05% Tween 20, the membrane was incubated with secondary antibodies for two hours at room temperature. After three additional washes, the protein bands were visualized using an ECL reagent kit (Solarbio, Catalog number: PE0010, Beijing, China) according to the manufacturer’s instructions.

### 4.7. RNA Extraction and RT-qPCR

To extract the total RNA, cells were harvested, and RNA was isolated using the RNAiso Plus reagent (Takara Bio, Shiga, Japan) following the manufacturer’s protocol. The concentration and purity of RNA were measured using NanoDrop 2000 (Thermo Scientific, Wilmington, DE, USA). A total of 2 μg of RNA was reverse transcribed using a PrimeScript RT Master Mix (Takara Bio, Japan). A real-time quantitative polymerase chain reaction (RT-qPCR) was carried out to determine the mRNA levels on a LightCycler96 real-time fluorescence qPCR instrument (Roche, Basle, Switzerland). The reaction mix (10 µL) consisted of forward and reverse primers (0.5 μL each), 3 μL of sterile deionized distilled water, 5 μL of SYBR Green Premix (Accubate Biology, Changsha, China), and cDNA templates (1 μL). GAPDH was used as an internal control. The primer sequences for the target genes (CDKN1A, FOXO1, and MELK) and GAPDH were as follows: GAPDH: forward: 5′-GTCATCATCTCTGCTCCTTCTG-3′, reverse: 5′-GCTGACAATCTTGAGGGAGTT-3′; CDKN1A: forward: 5′-ATCCCTCATGGCAGCAAG-3′, reverse: 5′-CTCGGTGACGAAGTCAAAGT-3′; FOXO1: forward: 5′-ATTCACCCAGCCCAAACTAC-3′, reverse: 5′-GAGTCCTGGTGCACAGTTATAC-3′; MELK: forward: 5′- GTGCTAGAGACAGCCAACAA-3′, reverse: 5′-AGGCGATCCTGGGAAATTATG-3′. All reactions were performed in triplicate. The relative expression was calculated using the 2^−ΔΔCt^ method.

### 4.8. Molecular Docking

The 3D structure of the MELK protein was downloaded from the PDB database (http://www.rcsb.org/, accessed on 1 March 2023) with the PDB code 4UMQ. AutoDock Tools (version 1.5.6) was used for protein isolation and modification. The docking analysis of the prepared ligands and target proteins was performed using Autodock Vina (The Scripps Research Institute, La Jolla, CA, USA). The molecular library ligands were placed into previously identified binding sites of the target protein using a grid box, and the docking binding energy was predicted. The docking results were visualized using Pymol 1.3 (DeLano Scientific, San Carlos, CA, USA).

### 4.9. Statistical Analysis

Graph Pad Software 9.0 (San Diego, CA, USA) was used to perform Dunnett’s test to determine the statistical significance. The results were presented as the means ± SEM, and the *p*-value less than 0.05 were considered significant.

## Data Availability

The RNA-Seq data were deposited in GEO (GSE232189).

## Supplementary Materials

The following supporting information can be downloaded at: https://www.mdpi.com/article/10.3390/molecules28145398/s1, Figure S1. Coptisine inhibits H1N1 virus infection by targeting MELK.
