# Supplementary material for: Coptisine Inhibits Influenza Virus Replication by Upregulating p21
Source: Molecules. 2023 Jul 14;28(14):5398. doi: 10.3390/molecules28145398 (PMC10386263; doi:10.3390/molecules28145398)
Supplement: Supplementary file 1 [file molecules-28-05398-s001.zip › molecules-2432614-supplementary.pdf]

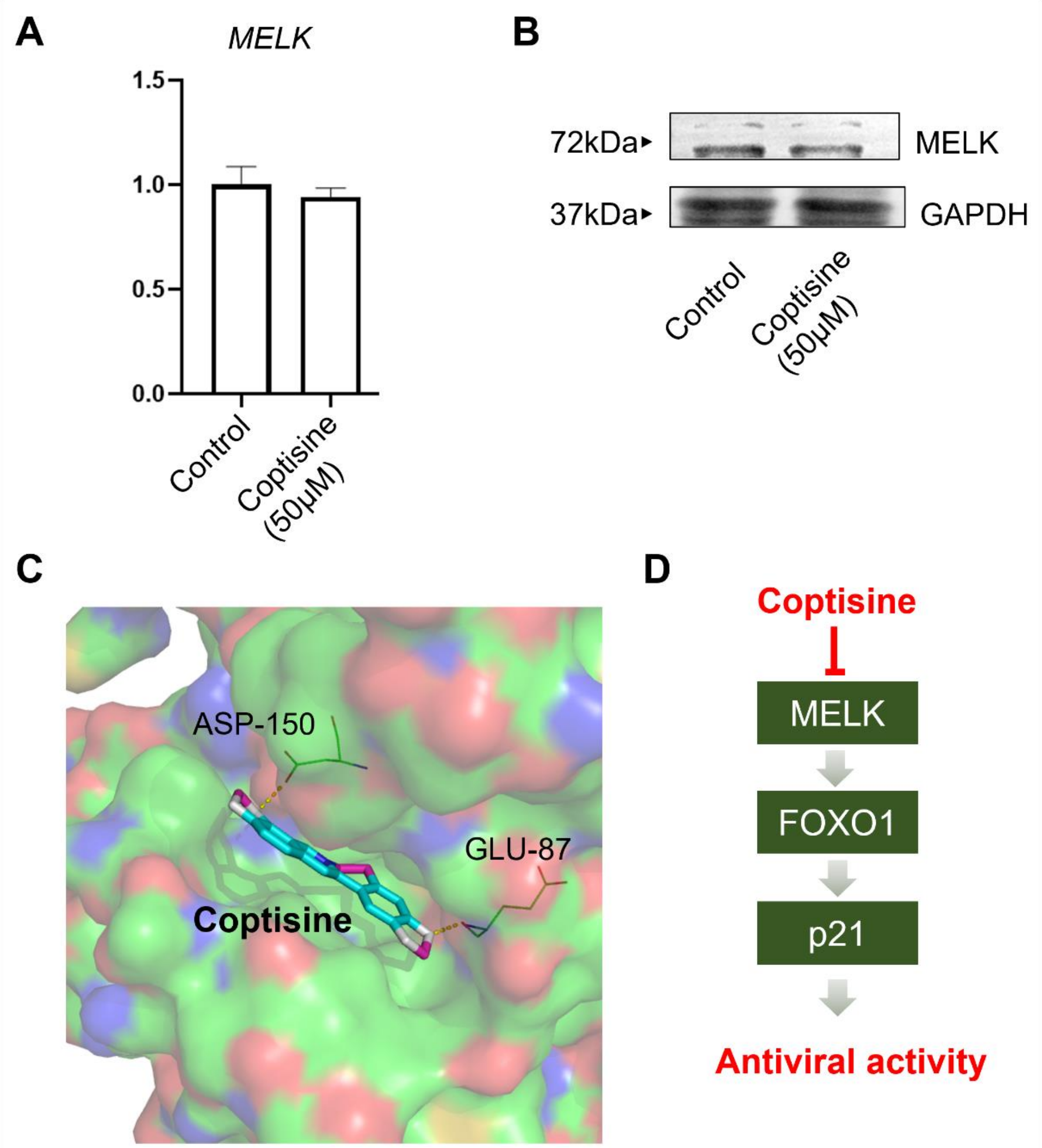

Figure S1. Coptisine inhibits H1N1 virus infection by targeting MELK. (**A**,**B**) MDCK was cultured for 24 h in the absence or in the presence of coptisine (50 μM). The total RNA was collected for reverse transcription and quantitative PCR measured (**A**). The total protein was also collected for western blot to quantify MELK protein levels (**B**). (**C**) The predicted binding model illustrates the favorable binding positions of coptisine (cyan stick) with the lowest binding free energy in the binding pocket of MELK. The key amino residues are labeled with green line and H-bond interactions are depicted as red dashes. (**D**) A schematic diagram of the role of coptisine in suppressing influenza virus infection by targeting MELK-mediated signaling pathway.
